# Novel DNA Variants and Mutation Frequencies of *hMLH1* and *hMSH2* Genes in Colorectal Cancer in the Northeast China Population

**DOI:** 10.1371/journal.pone.0060233

**Published:** 2013-04-03

**Authors:** Fulan Hu, Dandan Li, Yibaina Wang, Xiaoping Yao, Wencui Zhang, Jing Liang, Chunqing Lin, Jiaojiao Ren, Lin Zhu, Zhiwei Wu, Shuying Li, Ye Li, Xiaojuan Zhao, Binbin Cui, Xinshu Dong, Suli Tian, Yashuang Zhao

**Affiliations:** 1 Department of Epidemiology, Public Health College, Harbin Medical University, Harbin, People’s Republic of China; 2 Department of Colorectal Surgery, Cancer Hospital of Harbin Medical University, Harbin, People’s Republic of China; 3 Department of Surgery, The Fourth Affiliated Hospital, Harbin Medical University, Harbin, People’s Republic of China; The Chinese University of Hong Kong, Hong Kong

## Abstract

Research on *hMLH1* and *hMSH2* mutations tend to focus on Lynch syndrome (LS) and LS-like colorectal cancer (CRC). No studies to date have assessed the role of *hMLH1* and *hMSH2* genes in mass sporadic CRC (without preselection by MSI or early age of onset). We aimed to identify novel *hMLH1* and *hMSH2* DNA variants, to determine the mutation frequencies and sites in both sporadic and LS CRC and their relationships with clinicopathological characteristics of CRC in Northeast of China. 452 sporadic and 21 LS CRC patients were screened for germline and somatic mutations in *hMLH1* and *hMSH2* genes with PCR–SSCP sequencing. We identified 11 *hMLH1* and seven *hMSH2* DNA variants in our study cohort. Six of them were novel: four in *hMLH1* gene (IVS8-16 A>T, c.644 GAT>GTT, c.1529 CAG>CGG and c.1831 ATT>TTT) and two in *hMSH2* gene (−39 C>T, insertion AACAACA at c.1127 and deletion AAG at c.1129). In sporadic CRC, germline and somatic mutation frequencies of *hMLH1*/*hMSH2* gene were 15.59% and 17.54%, respectively (*p* = 0.52). Germline mutations present in *hMLH1* and *hMSH2* genes were 5.28% and 10.78%, respectively (*p*<0.01). Somatic mutations in *hMLH1* and *hMSH2* genes were 6.73% and 11.70%, respectively (*p = *0.02). In LS CRC, both germline and somatic mutation frequencies of *hMLH1*/*hMSH2* gene were 28.57%. The most prevalent germline mutation site in *hMSH2* gene was c.1168 CTT>TTT (3.90%), a polymorphism. Somatic mutation frequency of *hMLH1*/*hMSH2* gene was significantly different in proximal, distal colon and rectal cancer (*p* = 0.03). Our findings elucidate the mutation spectrum and frequency of *hMLH1* and *hMSH2* genes in sporadic and LS CRC, and their relationships with clinicopathological characteristics of CRC.

## Introduction

Colorectal cancer (CRC) is one of the most common malignancies globally, and ranks the fifth of all cancers in China. World Health Organization estimates that 220,000 new CRC cases occurred in China in 2008 (GLOBOCAN, 2008). The incidence of CRC has increased by 5.73% on a yearly basis between 1992 to 2005 (13.06 to 23.54/10,0000) in Nangang District, Harbin, China [1].

One of the genetic pathways in the development of CRC is the failure of DNA mismatch repair (MMR) system [2], which contributes to the maintenance of genomic stability by recognizing and removing insertion/deletion mutations that occur during DNA replication [3]. The two main mismatch repair genes are *hMLH1* and *hMSH2,* which map to chromosomes 3p21.3–23 [4] and 2p21–22 [5], respectively.

Since the first report of *hMLH1* and *hMSH2* gene mutations in Lynch syndrome (LS) CRC [4], [5], studies on *hMLH1* and *hMSH2* gene mutations have been published. However, the majority of the published papers focused on LS or LS-like CRC. In total, 30 small-sample size (n = 5–61, except for one of 315 patients) studies have been published that screened germline mutations in *hMLH1* and *hMSH2* genes in sporadic CRC. Pathological mutations of *hMLH1* and *hMSH2* genes were more likely to be present in younger patients [6], and in those with microsatellite instability (MSI). In our analysis of these 30 studies, MSI or early-age onset (under the age of 40, 45, 50 or 55 years) was used to preselect patients for *hMLH1* and *hMSH2* gene mutations in sporadic CRC. However, no study aimed to detect mutation frequencies of *hMLH1* and *hMSH2* genes in mass sporadic CRC without MSI or age preselection. In China, four studies (n = 26–58) screened germline or somatic mutations of *hMLH1* and *hMSH2* genes in sporadic CRC with preselection by MSI [7], [8], [9], [10]. Whether high frequencies of *hMLH1* and *hMSH2* gene mutations occur in sporadic CRC in China has not been elucidated. Moreover, strong evidence suggests that rare mutations of severe effect are responsible for a substantial portion of complex human cancer [11]. We therefore conducted this study to identify novel *hMLH1* and *hMSH2* DNA variants, to determine both the mutation frequencies and sites in both sporadic and LS CRC, and to estimate the relationships between germline and somatic mutations of *hMLH1/hMSH2* gene and clinicopathological characteristics of CRC in Northeast China.

## Materials and Methods

### Subjects

After obtaining informed consent from study subjects, and approval from Institutional Research Board of Harbin Medical University, we identified CRC patients who underwent surgery at the Cancer Hospital and the Second Affiliated Hospital of Harbin Medical University, without preselection and based on pathologic diagnosis alone. Patients with neuroendocrine carcinoma, malignant melanoma, non-Hodgkin’s lymphoma, gastrointestinal stromal tumors, and metastatic colorectal carcinoma were excluded from the analysis. From June 1, 2004 to May 15, 2005, and May 15, 2007 to January 1, 2008, 473 primary CRC patients (452 sporadic CRC; 21 LS CRC) were recruited. 457 blood samples and 356 tumor tissues were collected for molecular genetic analysis.

### DNA Extraction

DNA was successfully extracted from all 457 blood samples (436 sporadic CRC and 21 LS CRC) and 356 tumor tissues (342 sporadic and 14 LS) using the classical phenol-chloroform procedure [12].

In the collection of blood and tissue samples and DNA extraction, we could not obtain the tumor tissue DNA of 117 CRC patients (110 sporadic and 7 LS) due to that the tumor tissues were only big enough for pathology diagnosis or that we did not extract DNA successfully. Therefore, we only have their blood DNA. In the other 16 sporadic CRC patients, we obtained paired blood and tissue samples. However, in the DNA extraction, we did not extract DNA successfully from blood sample. Finally, 340 CRC patients (326 sporadic and 14 LS) have paired blood and tissue DNA.

### Screening for Germline and Somatic Mutations of *hMLH1* and *hMSH2* Genes

#### PCR–SSCP sequencing analysis

The primers for 20 pairs of all 19 exons in the *hMLH1* gene and 17 pairs of all 16 exons in the *hMSH2* gene (Table 1), including exon-intron boundaries, were synthesized for genomic PCR. PCR amplifications were performed using the following protocol for 35 cycles: denaturation for 30 s at 95°C, annealing for 30 s at 54°C to 64°C, extension for 30 s at 72°C, followed by a final extension for 5 min at 72°C (ABI 9700). PCR products were identified by 1% agarose electrophoresis (Biowest Agarose, Gene Company Ltd).

**Table 1:** Primers for *hMLH1* and *hMSH2* genes.

	Forwardoligonucleotide (5′–3′)Reverseoligonucleotide (5′–3′)	Annealingtemperature(°C)	Productsize(bp)
**hMLH1**			
Exon 1	AGGCACTTCCGTTGAGCATC GTAGCCCTTAAGTGAGCCCG	60	205
Exon 2	GTTTGATTTGCCAGTTTAGATG GTGCCCAGCAAATAATAGGTA	60	265
Exon 3	CTCATCTTTTTGGTATCTAACAG TCTTTAGCTTACCTCACCTCG	62	134
Exon 4	CTTTGGTGAGGTGACAGTGG GACAGGATTACTCTGAGACC	62	222
Exon 5	ATTAGAGCAAGTTACTCAGATG TATTACCCTGAAAACTTAGAAGC	62	173
Exon 6	GCTTTTGCCAGGACATCTTGG CAGAGACCCACTCCCAGATT	64	199
Exon 7&8	AGGTATTCAGTACACAATGCAG TTATATAGGTTATCGACATACC	60	303
Exon 9	CAGGAGGACCTCAAATGGACC GTTGATGAAGAGTAAGAAGATGC	62	261
Exon 10	ACCTTTCTTCCTGGGGATGTGAT GTTCCTTGTGAGTCTTGGTTGAG	64	278
Exon 11	GATCCTGAGGTTTTGACCACTG TGGATGAGAAGCGCCCTGACCT	62	300
Exon 12A	TACAGACTTTGCTACCAGGACT CTCTGTGACAATGGCCTGGG	64	209
Exon 12B	CTCTGTGACAATGGCCTGGG CAGAGGGCAAGTCAGGCAGAG	66	303
Exon 13	GTTGCTTGCTCCTCCAAAATGC CTTGGCAGTTGAGGCCCTATG	64	292
Exon 14	TTCTTTGCTTACTTGGTGTC TGGACCATTGTTGTAGTAGC	58	272
Exon 15	GGGTAAGAGATTTTGTTAGACTG TACCGATAACCTGAGAACACC	60	247
Exon 16	TCCTTCATGTTCTTGCTTCT GCTGTCACACCTCATCAAT	58	201
Exon 17	GCCTGGGAAAGCACTGGAGA ACCGAAATGCTTAGTATCTGCT	64	211
Exon 18	GTAGTCTGTGATCTCCGTT ATTGTATGAGGTCCTGTCC	56	245
Exon 19A	CAAACAGGGAGGCTTATGAC CGGAATACAGAGAAAGAAGAAC	64	256
Exon 19B	GCTTGCTAACCTGCCTGAT CAATCCACTGTGTATAAAGG	58	208
**hMSH2**			
Exon 1	CGGGAAACAGCTTAGTGGGT GGCCCCATGTACTTGATCAC	64	272
Exon 2	TCTCGGGTATGTCTTTATCAGC CCTTATATGCCAAATACCAATC	60	253
Exon 3	AGGCTTCTCCTGGCAATCTCT TTTCCCCATGTCTCCAGCAGT	66	274
Exon 4	CTTATTCCTTTTCTCATAGTAG TCCATGTACCTGATTCTCC	60	202
Exon 5	ATCCAGTGGTATAGAAATC CCTTTATAAGCTTCTTCAGT	56	289
Exon 6	TGTTTTTCATGGCGTAGTAAGG TACCTCTCCTCTATTCTGTTCT	62	209
Exon 7	TCAGATTGAATTTAGTGGAAGC TTCATGTTTTTCCAGAGCCTG	55	203
Exon 8	GATGCTTGTTTATCTCAGTC CTGTCCACAAAGGTGCTAC	58	313
Exon 9	CTGAATAACTTATGGATAGC TCCAACCTCCAATGACCCA	60	279
Exon 10	TGGTAGTAGGTATTTATGG CATCATGTTAGAGCATTTAG	58	263
Exon 11	TGTTTCATAGGATACTTTGG CCAGGTGACATTCAGAACATT	60	235
Exon 12	CAGGCTATGTAGAACCAATGC CCACAAAGCCCAAAAACCAG	64	278
Exon 13	TAGGCCCCAATATGGGAGGT AAGCAGTTTCCAACATTTCAGC	60	198
Exon 14	ATTATGTGCTTCAGGTCTGC GTACATACCTTTCTTCACCTGAT	52	270
Exon 15	ATGCTGTCCCCTCACGCTTC AGCACTTCTTTGCTGCTGGTTC	61	198
Exon 16A	TCAGGAGTTCCTGTCCAAGG TTACCTTCATTCCATTACTGGG	57	181
Exon 16B	TCCCAGTAATGGAATGAAGGT CACTGCGAAGAACTACAATGC	64	211

PCR products were denatured at 98°C for 8 min and placed on ice. Electrophoresis was performed on 8% to 15% nondenaturing polyacrylamide gels. After electrophoresis, gels were stained with silver (Refined Chemical Plant, Shanghai, China). 15% of the samples were replicated in detecting mutations of every amplified PCR fragment in the PCR-SSCP analysis, with the concordance rate ranging from 99.1% to 100% for various amplified PCR fragments.

PCR products showing abnormal mobility under SSCP analysis were sent to sequence using ABI3730XL. Sequencing results were analyzed for gene mutations with Chromas 2.22 software (Technelysium Pty. Ltd., QLD, Australia).

### Assessment of Mutation Pathogenicity

For previously reported mutations, results of function verification were used to determine pathogenicity. If no function verification was reported, function prediction by any two of the PolyPhen/SIFT/MAPP-MMR results was used to determine their pathogenicity.

For the novel DNA variants, the pathogenicity of base substitution in exons were predicted by PolyPhen program [13] and MAPP-MMR [14]. Base insertion, deletion and substitution in promoter, introns or 3′UTR were assessed by criteria to determine potential pathogenicity [15]. We also detected the novel DNA variants in 100 healthy controls to determine potential pathogenicity.

### Statistical Analysis

Category and continuous variables were tested by the chi-square test and *t* test, respectively. All the statistical analyses were performed by SAS 9.1 (SAS Institute, Cary, NC, USA).

## Results

### Mutations

#### Mutations in *hMLH1* gene

**Figure 1:**
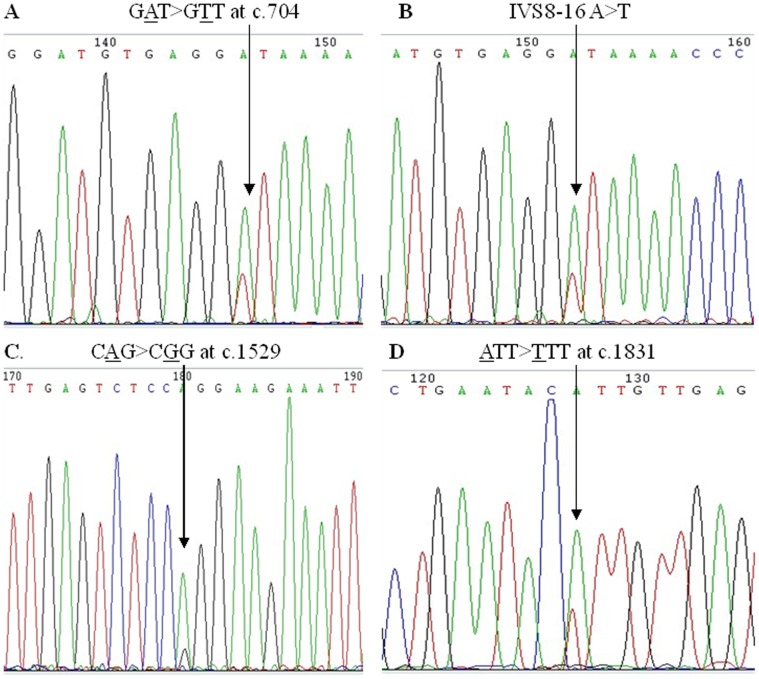
Four novel DNA variants in *hMLH1* gene.

We identified 11 DNA variants in *hMLH1* gene. IVS8-16 A>T, c.1831 ATT>TTT and c.1845_1847 deletion GAA were somatic DNA variants, other eight DNA variants were both germline and somatic variants. Four (IVS8-16 A>T, c.704 GAT>GTT, c.1529 CAG>CGG, c.1831 ATT>TTT) were novel DNA variants identified in sporadic CRC patients (Figure 1 and Table 2). All the four novel DNA variants were not detected in 100 healthy controls. c.1529 CAG>CGG was predicted to have no pathogeneity, the pathogeneity of other three novel DNA variants were uncertain. Seven mutations (−28 A>G, c.927 CCC>CCT, IVS13+14 G>A, IVS14-19 A>G, c.1742 CCG>CTG, c.1845_1847 deletion GAA and c.*35_*37 deletion CTT) were previously reported in the InSiGHT database [10], [16], [17], [18], [19], [20], [21]. c.1742 CCG>CTG and c.1845_1847 deletion GAA were reported to be pathologic mutations [21], [22].

**Table 2:** DNA variants found in *hMLH1* and *hMSH2* genes.

Mutation/location	Nucleotide change	Amino acid change	Mutation type	Polyphen†	MAPP-MMR††	Pathogeneity	Characteristics of patients carried novel DNA variant
**Novel DNA variants**							
*hMLH1*/Intron 8	IVS8-16 A>T	–	–	–	–	Uncertain	51 years, ulcerated adenocarcinoma, moderately differentiation, Dukes B, tumor size: 6*4.5*1 cm^3^
*hMLH1*/Exon 9	c.704 GAT>GTT	p.Asp235Val	Missense	0.052	6.710	Uncertain	56 years, protrude type adenocarcinoma, moderately differentiation, Dukes B, tumor size: 5*5*3 cm^3^
*hMLH1*/Exon 13	c.1529 CAG>CGG	p.Gln510Arg	Missense	0.002	1.380	No	71 years, ulcerated and infiltrating adenocarcinoma, poorly differentiation, Dukes B, tumor size: 7*8*9 cm^3^
*hMLH1*/Exon 16	c.1831 ATT>TTT	p.Ile611Phe	Missense	0.125	2.680	Uncertain	51 years, ulcerated infiltrating adenocarcinoma, moderately differentiation, Dukes C, tumor size: 5*5*6 cm^3^
*hMSH2*/5′UTR	−39 C>T	–	–	–	–	Uncertain	–‡
*hMSH2*/Exon 7*	c.1127 ins AACAACA, c.1129 del AAG	Out of frame insertion in codon376, In-framedeletion in codon377	Frameshift	–	–	Uncertain	protrude type myxo-adenocarcinoma, poorly-moderately differentiation, Dukes C, tumor size: 4*4*5 cm^3^
**Previously reported mutations**							
*hMLH1*/5′UTR	−28 A>G	–	–	–	–	Unknown [45], [46]	–
*hMLH1*/Exon 11	c.927 CCC>CCT	p.Pro309Pro	Synonymous	–	–	Unknown [19]	–
*hMLH1*/Intron 13	IVS13+14 G>A	–	–	–	–	Unknown [45]	–
*hMLH1*/Intron 14	IVS14-19 A>G	–	–	–	–	Unknown [47], [48]	–
*hMLH1*/Exon 16	c.1742 CCG>CTG	p.Pro581Leu	Missense	–	–	Reported pathogenicity[14], [21]	–
*hMLH1*/Exon 16	c.1845_1847 del GAA	In-frame deletion in codon615_616	No change	–	–	Reported pathogenicity[10], [49]	–
*hMLH1*/3′ UTR	*35_*37 del CTT	–	–	–	–	Unknown [18], [50]	–
*hMSH2*/Exon 1	c.23 ACG>ATG	p.Thr8Met	Missense	–	–	No [14], [51]	–
*hMSH2*/Exon 3	c.471 GGC>GGA	p.Gly157Gly	Synonymous	–	–	No [27]	–
*hMSH2*/Exon 3	c.505 ATA>GTA	p.Ile169Val	Missense	–	–	Unknown [14], [45]	–
*hMSH2*/Exon 7	c.1168 CTT>TTT	p.Leu390Phe	Missense	–	–	Unknown [8], [14]	–
*hMSH2*/Exon 12	c.1886 CAA>CGA	p.Gln629Arg	Missense	–	–	No [8], [45]	–

* DNA variant found in the relapse tumor of a LS CRC patient.

‡ The variants were detected in more than one patient, so we did not describe the characteristics of these patients.

† If PolyPhen score >2.0 then the AA substitution is predicted to affect protein function.

†† If MAPP-MMR score >4.55 then the AA substitution is predicted to affect protein function.

We also identified two polymorphisms. c.655 ATC>GTC was reported to be a common polymorphism in Caucasians [23], [24], [25], while c.1151 GTT>GAT was reported to be more common in Asian population [26]. Therefore, we did not categorize them as mutations in our study.

#### Mutations in *hMSH2* gene

**Figure 2:**
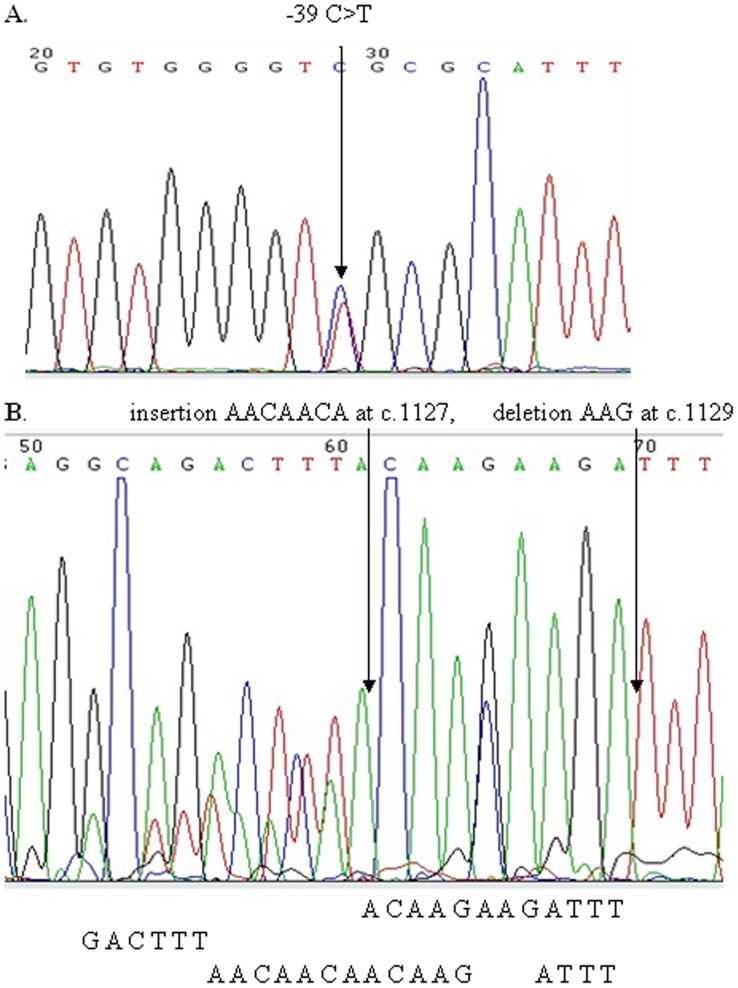
Two novel DNA variants in *hMSH2* gene.

We identified seven *hMSH2* DNA variants. Insertion AACAACA at c.1127 and deletion AAG at c.1129 was somatic DNA variants, other six DNA variants were both germline and somatic variants. Two DNA variants (−39 C>T, insertion AACAACA at c.1127 and deletion AAG at c.1129) were newly detected in this study (Figure 2 and Table 2). In screening the two novel DNA variants in 100 healthy controls, no variants were detected. The pathogenicity of the two DNA variants was uncertain. Five other mutations (c.23 ACG>ATG, c.471 GGC>GGA, c.505 ATA>GTA, c.1168 CTT>TTT and c.1886 CAA>CGA) were previously reported in the InSiGHT database [14], [27].

Two male patients carried somatic mutations in both *hMLH1* and *hMSH2* genes. Another male patient carried the c.1831 ATT>TTT mutation of the *hMLH1* gene and the c.23 ACG>ATG mutation of the *hMSH2* gene in both tumor tissues and blood.

### Mutation Frequencies

#### Mutation frequencies in sporadic CRC patients

Among 436 sporadic CRC patients with available blood DNA, germline mutation frequencies of *hMLH1* and *hMSH2* genes were 5.28% (23/436) and 10.78% (47/436), respectively (*p*<0.01) (Table 3). Excluding the synonymous mutations (c.927 CCC>CCT in *hMLH1* and c.471 GGC>GGA in *hMSH2*), the mutation frequencies in *hMLH1* and *hMSH2* genes were 4.59% (20/436) and 8.72% (38/436), respectively (*p = *0.01). If the patient who carried two germline mutations was only counted once (one patient harbored both −39 C>T and c.23 ACG>ATG mutations in *hMSH2* and the IVS13+14 G>A mutation of *hMLH1*; the other patient carried both c.1831 ATT>TTT mutation in *hMLH1* and c.23 ACG>ATG in *hMSH2*); then 15.59% (68/436) patients exhibited germline mutations in *hMLH1*/*hMSH2* gene. Pathologic mutation frequencies of *hMLH1* and *hMSH2* genes were 0.23% (1/436) and 0%, respectively.

**Table 3:** Mutation frequencies of *hMLH1* and *hMSH2* genes.

Mutation/location	Somatic mutation frequency		Germline mutation frequency	
	Sporadic CRC	LS CRC	Sporadic CRC	LS CRC
***hMLH1***				
*hMLH1*/5′UTR	0	7.14% (1/14)	0	4.76% (1/21)
*hMLH1*/Intron 8	0.29% (1/342)	–	–	–
*hMLH1*/Exon 9	0.29% (1/342)	0	0.23% (1/436)	0
*hMLH1*/Exon 11	0.88% (3/342)	0	0.69% (3/436)	0
*hMLH1*/Intron 13	1.46% (5/342)	0	1.38% (6/436)	0
*hMLH1*/Intron 14	2.63% (9/342)	0	2.52% (11/436)	0
*hMLH1*/Exon 16	0.88% (3/342)	0	0.23% (1/436)	0
*hMLH1*/3′UTR	0.29% (1/342)	0	0.23% (1/436)	0
Subtotal	6.73% (23/342)	7.14% (1/14)	5.28% (23/436)	4.76% (1/21)
***hMSH2***				
*hMSH2*/5′UTR††	0.58% (2/342)	0	0.46% (2/436)	0
*hMSH2*/Exon 1	2.63% (9/342)	0	1.38% (6/436)	0
*hMSH2*/Exon 3	1.75% (9/342)	7.14% (1/14)	2.06% (11/436)	4.76% (1/21)
*hMSH2*/Exon 7	4.39% (15/342)	7.14% (1/14)	3.90% (17/436)	14.29% (3/21)†
*hMSH2*/Exon 12	1.46% (5/342)	7.14% (1/14)	2.52% (11/436)	4.76% (1/21)
Subtotal	11.70% (40/342)	21.43% (3/14)	10.78% (47/436)	23.81% (5/21)
Total	17.54% (60/342)‡	28.57% (4/14)	15.59% (68/436)‡	28.57% (6/21)

† The mutation was also found in another blood sample of LS relapsed CRC patient.

†† One patient carried both –39 C>T and c.23 ACG>ATG.

‡ Three patients carried somatic mutations in both *hMLH1* and *hMSH2* genes; one patient carried germline mutations in both *hMLH1* and *hMSH2* genes.

Among 342 sporadic CRC patients with available DNA in tumor tissues, the somatic mutation frequencies in *hMLH1* and *hMSH2* genes were 6.73% (23/342) and 11.70% (40/342), respectively (*p = *0.02) (Table 3). Excluding synonymous mutations (c.927 CCC>CCT in *hMLH1* and c.471 GGC>GGA in *hMSH2*), mutation frequencies of *hMLH1* and *hMSH2* genes were 5.85% (20/342) and 9.94% (34/342), respectively (*p = *0.03). If mutations were counted by patients instead of the actual number of mutations (three patients carried somatic mutations of both *hMLH1* and *hMSH2* genes), then 17.54% (60/342) patients exhibited somatic mutations of *hMLH1*/*hMSH2* gene. Pathological mutation frequencies of *hMLH1* and *hMSH2* genes were 0.58% (2/342) and 0%, respectively.

Germline mutation frequency was not significantly different from that of somatic mutation frequency in *hMLH1* and *hMSH2* genes, respectively (*p = *0.49 and *p = *0.69, respectively).

#### Mutation frequencies in LS CRC patients

Among 21 blood DNA samples of LS CRC patients, one (4.76%) patient carried a germline mutation of *hMLH1* and five (23.81%) patients carried germline mutations in *hMSH2*. Overall, six (28.57%) patients exhibited germline mutations of the *hMLH1*/*hMSH2* gene.

Tumor tissues were only available in 14 LS CRC patients, one (7.14%) patient carried a somatic mutation in *hMLH1* and three (21.43%) patients carried a somatic mutation in *hMSH2*. In total, four (28.57%) patients exhibited somatic mutations in *hMLH1*/*hMSH2* gene.

No pathologic mutations were detected in LS CRC patients.

### The Mutation Distribution in Different Exons

The highest germline mutation prevalence of *hMSH2* in sporadic CRC was detected in exon 7 (3.90%), followed by exon 12 (2.52%), exon 1 (1.38%), and exon 3 (0.46%). Mutations in these four exons accounted for 76.6% of the total mutations in *hMSH2*. As far as the *hMLH1*, mutation frequencies were generally lower than in *hMSH2*; the highest mutation prevalences were in exon 16, exon 9, exon 13, and exon 19 (0.23%) (Table 3).

### The Relationships between Germline and Somatic Mutations of *hMLH1/hMSH2* Gene and Clinicopathological Characteristics of CRC

Somatic mutation frequency of *hMLH1*/*hMSH2* gene was 22.7% (15/66) in proximal colon cancer, 17.7% (11/62) in distal colon cancer and 10.5% (22/209) in rectal cancer (*p* = 0.03). Whereas, germline mutation frequency of *hMLH1*/*hMSH2* gene was not significantly different in proximal colon cancer (17.3%, 14/81), distal colon cancer (17.8%, 13/73) and rectal cancer (10.1%, 28/276) (*p* = 0.09). (Table 4 and 5).

**Table 4:** The relationships between germline mutation of *hMLH1/hMSH2* gene and clinicopathological features of the 436 sporadic CRC patients.

	No. of patients 436 (%)	Germline mutated 56 (%)	Wild type 380 (%)	*P* value
**Age(yr) at CRC diagnosis**				0.59
Mean	58.72 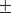 11.30	58.55 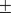 11.54	58.72 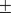 11.27	
				0.98
<40	27 (6.0)	3 (5.4)	23 (6.1)	
40–60	187 (43.0)	24 (42.9)	163 (43.0)	
≥60	222 (51.0)	29 (51.8)	193 (50.9)	
**Gender**				0.21
Male	262 (60.2)	38 (67.9)	224 (59.1)	
Female	173 (39.8)	18 (32.1)	155 (40.9)	
**BMI**				0.13
< = 21	107 (25.5)	15 (27.8)	92 (25.2)	
>21 and< = 25	182 (43.4)	17 (31.5)	165 (45.2)	
>25	130 (31.1)	22 (40.7)	108 (29.6)	
**Location**				0.09
Proximal colon cancer	81 (18.8)	14 (25.5)	67 (17.9)	
Distal colon cancer	73 (17.0)	13 (23.6)	60 (16.0)	
Rectal cancer	276 (64.2)	28 (50.9)	248 (66.1)	
**Dukes stage**				0.72
1	46 (10.6)	7 (12.5)	39 (10.3)	
2	190 (43.8)	23 (41.1)	167 (44.2)	
3	161 (37.1)	23 (41.1)	138 (36.5)	
4	37 (8.5)	3 (5.4)	34 (9.0)	
				0.90
1+2	236 (54.4)	30 (53.6)	205 (54.5)	
3+4	198 (45.6)	26 (46.4)	172 (45.5)	
**Histotypes**				0.52
Adenocarcinoma	332 (76.3)	46 (82.1)	286 (75.5)	
Mucinous adenocarcinoma	78 (17.9)	8 (14.3)	70 (18.5)	
Others	25 (5.7)	2 (3.6)	23 (6.1)	
**Pathological types**				0.83
Protrude type	273 (66.4)	33 (63.5)	240 (66.9)	
Ulceration type	30 (7.3)	4 (7.7)	26 (7.2)	
Ulceration+Infiltrating type	94 (22.9)	14 (26.9)	80 (22.3)	
Infiltrating type	14 (3.4)	1 (1.9)	13 (3.6)	
**Differentiated degree**				0.33
Poor	74 (17.9)	6 (18.9)	68 (18.9)	
Moderate	335 (81.1)	46 (86.8)	289 (80.3)	
Well	4 (1.0)	1 (1.9)	3 (0.8)	
**Tumor size**				0.38
< = 50	186 (47.9)	21 (41.2)	165 (49.0)	
50< and < = 200	141 (36.3)	23 (45.1)	118 (35.0)	
>200	61 (15.7)	7 (13.7)	54 (16.0)	

**Table 5:** The relationships between somatic mutation of *hMLH1/hMSH2* gene and clinicopathological features of the 342 sporadic CRC patients.

	No. of patients 342 (%)	Germline mutated 50 (%)	Wild type 380 (%)	*P* value
**Age(yr) at CRC diagnosis**				0.22
Mean	58.79 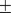 11.26	58.86 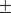 12.08	58.75 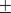 11.15	
				0.98
<40	20 (5.9)	3 (6.0)	17 (5.8)	
40–60	148 (43.4)	21 (42.0)	127 (43.6)	
≥60	173 (50.7)	26 (52.0)	147 (50.5)	
**Gender**				0.22
Male	205 (60.1)	34 (68.0)	171 (58.8)	
Female	136 (39.9)	16 (32.0)	120 (41.2)	
**BMI**				0.63
< = 21	86 (25.8)	14 (29.2)	72 (25.3)	
>21 and< = 25	146 (43.8)	18 (37.5)	128 (44.9)	
>25	101 (30.3)	16 (33.3)	85 (29.8)	
**Location**				0.03
Proximal colon cancer	66 (19.6)	15 (31.2)	51 (17.6)	
Distal colon cancer	62 (18.4)	11 (22.9)	51 (17.6)	
Rectal cancer	209 (62.0)	22 (45.8)	187 (64.7)	
**Dukes stage**				0.94
1	33 (9.7)	4 (8.0)	29 (10.0)	
2	154 (45.4)	22 (44.0)	132 (45.7)	
3	131 (38.6)	21 (42.0)	110 (38.1)	
4	21 (6.2)	3 (6.0)	18 (6.2)	
				0.63
1+2	187 (55.2)	26 (52.0)	161 (55.7)	
3+4	152 (44.8)	24 (48.0)	128 (44.3)	
**Histotypes**				0.97
Adenocarcinoma	262 (76.8)	39 (78.0)	223 (76.6)	
Mucinous adenocarcinoma	66 (19.4)	9 (18.0)	57 (19.6)	
Others	13 (3.8)	2 (4.0)	11 (3.8)	
**Pathological types**				0.93
Protrude type	216 (67.3)	31 (66.0)	185 (67.5)	
Ulceration type	21 (6.5)	4 (8.5)	17 (6.2)	
Ulceration+Infiltrating type	75 (23.4)	11 (23.4)	64 (23.4)	
Infiltrating type	9 (2.8)	1 (2.1)	8 (2.9)	
**Differentiated degree**				0.74
Poor	54 (16.6)	7 (15.2)	47 (16.8)	
Moderate	268 (82.5)	39 (84.8)	229 (82.1)	
Well	3 (0.9)	0 (0.0)	3 (1.1)	
**Tumor size**				0.10
< = 50	132 (43.7)	15 (32.6)	117 (45.7)	
50< and < = 200	121 (40.1)	25 (54.3)	96 (37.5)	
>200	49 (16.2)	6 (13.0)	43 (16.8)	

Germline and somatic mutation frequency of *hMLH1*/*hMSH2* gene was not significantly different in other clinicopathological characteristics (age, gender, BMI, Dukes stage, Histotypes, Pathological types, Differentiated degree and tumor size) of CRC.

Because of less LS CRC patients, we did not analyze the relationships between germline and somatic *hMLH1/hMSH2* gene mutations and clinicopathological characteristics of LS CRC.

## Discussion

Under the supposed model of common disease-rare variant [28], [29], we screened the rare variants of *hMLH1* and *hMSH2* genes in sporadic and LS CRC. We identified 18 types of DNA Variants in our study. Six were novel DNA variants and 12 have been previously reported. Of the six novel DNA variants, four were in *hMLH1* and two in *hMSH2*.

Two of the four novel *hMLH1* DNA variants, p.Asp235 Val (c.644 GAT>GTT) and p.Gln510Arg (c.1529 CAG>CGG), both lead to amino acid polarity changes, which may affect the structure of the *hMSH2* binding domain and *hPMS2/hPMS1* binding domain of the *hMLH1* gene respectively and cause the dysfunction of DNA MMR system. Another DNA variance, p.Ile611Phe (c.1831 ATT>TTT), lead to no amino acid polarity changes in the *hPMS2/hPMS1* binding domain of the *hMLH1* gene product, may have no effect on the function of DNA MMR system [30]. IVS8-16 A>T is predicted to have no effect on splicing in exon 9.

One of the two novel *hMSH2* DNA variants, −39 C>T, was a variance in 5′UTR, which may affect mRNA Transcription. The other variance, c.1127 ins AACAACA and c.1129 del AAG, was a frameshift mutation, which may affect the *hMSH6* binding domain and *hMutL* homolog interaction of the *hMSH2* gene product and cause the dysfunction in the DNA MMR system [30].

Although the failure of DNA MMR system is one of the genetic pathways in the development of CRC [2]. According to the criteria of mutation pathogeneity assessment, one novel DNA variant, c.1529 CAG>CGG, was predicted to have no pathogeneity, the pathogeneity of other five novel DNA variants were uncertain. Therefore, we cannot elucidate the role of these novel DNA variants of *hMLH1* and *hMSH2* genes in the occurrence and development of CRC.

c.1742 CCG>CTG of *hMLH1* and c.1886 CAA>CGA of *hMSH2* were founder mutations in the Asian population [31]. Three other mutations of *hMSH2*, c.23 ACG>ATG, c.505 ATA>GTA and c.1168 CTT>TTT, were of higher prevalence in Asians (2.44%, 1.74%, and 6.97%, respectively) compared with Caucasians (0.05%, 0.05%, and 0.53%, respectively) [31]. It may explain the racial difference of CRC patients. In addition, it may be more efficient to detect these mutations in Asian populations.

Since we detected a higher prevalence of c.1168 CTT>TTT of *hMSH2* in both LS (14.29%, 3/21) and sporadic (3.90%, 17/436) CRC, we screened for the mutation in healthy controls. The mutation frequency in healthy controls was 4.16% (21/505), which was not significantly different comparing with CRC (*p* = 0.84). This particular mutation was also reported as a polymorphism in Korea by Kim et al, who did not detect a significant difference between cases and controls [26].

Significant association was only observed between somatic *hMLH1/hMSH2* gene mutations and tumor location of sporadic CRC (*p = *0.03). The somatic mutation frequency of *hMLH1/hMSH2* gene was highest in rectal cancer, the following was in proximal colon cancer, and the lowest was in distal colon cancer. The non-pathogeneity or uncertain pathogeneity may explain the non-significant association between *hMLH1/hMSH2* gene mutations and other clinicopathological characteristics of sporadic CRC.

In our previous meta-analysis based on the germline mutations of *hMLH1* and *hMSH2* genes (paper accepted, 10.1371/journal.pone.0051240), the pooled pathologic mutation frequency of *hMLH1* was 8.72% (95%CI: 6.12%–12.29%) in sporadic CRC. It was 10.28% (95% CI: 4.28–22.70%) in American studies, 7.47% (95% CI: 4.06–13.34%) in European studies, and 3.21% (95% CI: 0.88–11.03%) in Asian studies (*p = *0.65). In our cohort, it was only 0.23%. The pooled pathologic mutation frequency of *hMSH2* was 7.28% (95% CI: 5.12%–10.26%) in sporadic CRC. It was 5.89% (95% CI: 2.08–15.61%) in American studies, 7.58% (95% CI: 4.05–13.76%) in European studies, and 3.64% (95% CI: 1.96–6.65%) in Asian studies (*p* = 0.85). However, no pathologic mutation of *hMSH2* was detected in our study.

Eight [7], [9], [32], [33], [34], [35], [36], [37] and nine studies [7], [9], [32], [33], [34], [35], [36], [37], [38] in Asia detected somatic mutations of *hMLH1* and *hMSH2* genes in sporadic CRC. The pooled prevalence of pathologic mutations was 11.86% (95% CI: 7.62–18.01%) and 7.90% (95% CI: 4.72–12.94%) respectively upon meta-analysis, which is higher than that in our study (0.58% and 0%).

All the published studies detected germline or somatic mutations in sporadic CRC with preselection (MSI, early-onset age, or TGF-β RII mutation) [36], which could explain the higher mutation frequency in the published individual studies and meta-analyses of previously published studies. In addition, the small sample size in those published studies may also contribute to the inconsistent results.

Only one study in Asia detected somatic mutations of *hMLH1* and *hMSH2* genes in 31 sporadic CRC patients without preselection [37]. The largest study detecting germline mutations was of 315 European BG-CRC patients under the age of 55; the mutation frequency of *hMSH2* was found to be 0.32% (1/325, uncertain pathogenicity), whereas no mutation in *hMLH1* was detected [39].

In our previous meta-analysis, the pooled germline mutation frequencies of *hMLH1* and *hMSH2* genes were 28.55% (95% CI: 26.04%–31.19%) and 19.41% (95% CI: 15.88%–23.51%) in Amsterdam-criteria positive LS CRC. In Amsterdam-criteria negative LS CRC, these pooled mutation frequencies were 16.70% (95% CI: 14.53–19.13%) and 11.13% (95% CI: 9.49–13.42%) for *hMLH1* and *hMSH2* genes, respectively. In our study, no germline mutation in *hMLH1* exons was found, similar to a study in Japan [40]. The germline mutation frequency of *hMSH2* was 9.52% (2/21) (excluding the polymorphic mutation c.1168 CTT>TTT), which was relatively lower than that in the meta-analysis (11.13%, 95% CI: 9.49–13.42%).

Five Asian studies detected the somatic mutation of *hMLH1* or *hMSH2* in LS CRC [7], [41], [42], [43], [44]. The pooled somatic mutation frequencies in *hMLH1* and *hMSH2* genes were 9.57% (95% CI: 1.36–44.73%) and 25.65% (95% CI: 10.30–50.89%), respectively upon meta-analysis. In our study, the somatic mutation frequency of *hMSH2* in LS CRC was 14.29% (2/14) (excluding the polymorphic mutation, c.1168 CTT>TTT). However, no somatic mutations in *hMLH1* exons were found in LS CRC, similar to the two Japanese studies [41], [43]. The somatic mutation frequency of *hMSH2* in LS CRC varied from 5.88% to 58.33% in the five Asian published studies. A small sample size may explain the variances of mutation frequency in LS CRC.

In conclusion, we identified six novel DNA variants (four in *hMLH1* and two in *hMSH2*). In sporadic CRC, germline and somatic mutation frequencies of *hMLH1/hMSH2* gene were 15.59% and 17.54%, respectively. The prevalence of germline mutations was 5.28% in *hMLH1* and 10.78% in *hMSH2*. The somatic mutation frequencies in *hMLH1* and *hMSH2* genes were 6.43% and 11.70%, respectively. In LS CRC, both germline and somatic mutation frequencies of *hMLH1*/*hMSH2* gene were 28.57%. The most prevalent germline mutation site in *hMSH2* gene was c.1168 CTT>TTT (3.90%), a polymorphism. Somatic mutation frequency of *hMLH1*/*hMSH2* gene was significantly different in proximal colon cancer, distal colon cancer and rectal cancer.

Our findings could help to elucidate the DNA variant spectrum and frequency of the *hMLH1* and *hMSH2* genes in CRC patients, especially sporadic CRC patients in China, and their relationships with clinicopathological characteristics of sporadic CRC. Functional studies to determine how these novel DNA variants affect protein function are required.
